# High expression of TMEM40 is associated with the malignant behavior and tumorigenesis in bladder cancer

**DOI:** 10.1186/s12967-017-1377-3

**Published:** 2018-01-19

**Authors:** Zhen-Fei Zhang, Han-Rong Zhang, Qing-Yan Zhang, Shu-Yu Lai, Yu-Zhen Feng, Yi Zhou, Si-Rong Zheng, Rong Shi, Jue-Yu Zhou

**Affiliations:** 1 Department of Biochemistry and Molecular Biology, School of Basic Medical Sciences, Southern Medical University, Guangzhou, 510515 People’s Republic of China; 2 Guangdong Provincial Key Laboratory for Biochip Technology, Guangzhou, 510515 People’s Republic of China; 3 The First School of Clinical Medicine, Southern Medical University, Guangzhou, People’s Republic of China; 4 Department of Biology Medicine and Advanced Materials Research Center, Shantou University, Shantou, 515063 Guangdong People’s Republic of China

**Keywords:** TMEM40, Bladder cancer, Malignant phenotype, Tumorigenesis, p53 pathway

## Abstract

**Background:**

Bladder cancer (BCa) is one of the most common cancers in the urinary system among the world. Previous studies suggested that TMEM40 expression level was significantly associated with clinicopathological parameters including histological grade, clinical stage and pT status of bladder cancer. However, the molecular mechanism of TMEM40 in BCa remains poorly understood.

**Methods:**

Real-time quantitative RT-PCR (qRT-PCR) and western blot (WB) were used to examine the expression levels of TMEM40 in BCa tissues, paired non-cancer tissues and cell lines. A series of experiments, including CCK-8, wound healing, flow cytometry, transwell and EdU assays were performed to assess the effects of TMEM40 on cell proliferation, cell cycle and apoptosis, migration and invasion. In addition, tumor growth was evaluated in vivo using a xenogenous subcutaneously implant model. All statistical analyses were executed by using the SPSS 20.0 software. All experimental data from three independent experiments were analyzed by Student’s *t* test and results were expressed as mean ± standard deviation.

**Results:**

In this study, we identified the role of TMEM40 in the tumorigenesis of bladder cancer and found that it was upregulated in bladder cancer tissues and cell lines, compared with their normal counterparts. The results demonstrated that effective silence of TMEM40 expression suppressed cell proliferation, blocked G1-to-S cell cycle transition, and inhibited cell migration and invasion in human bladder 5637 and EJ cell lines. Consistently, in vivo data showed that TMEM40 silencing could dramatically decreased tumor growth. Further study revealed that TMEM40 knockdown resulted in accumulation of p53 and p21 protein and decrease of c-MYC and cyclin D1 protein.

**Conclusion:**

These data suggest that TMEM40 represents a potential oncogene, which exert a crucial role in the proliferation and apoptosis via the p53 signaling pathway in BCa, thus probably serve as a novel candidate biomarker and a potential therapeutic target for patients with BCa.

## Background

Bladder cancer (BCa) is one of the most common urological malignancies across the world, especially in elderly men [1]. It is reported that there are 386,000 new cases and almost 150,000 deaths worldwide annually. BCa is classified as non-muscle invasive and muscle-invasive cancers [2]. Approximately, 70% of patients display indications for non-muscle-invasive BCa (NMIBC) during the initial diagnosis with mutable danger of recurrence and development to invasive diseases, hence needing long-term surveillance [3]. The recommended maintenance schedules from transurethral resection to radiation therapy and systemic chemotherapy at present are effective only in a subset of patients, and the 5-year overall survival rate remains at a low level [4].

Although numerous genes associated with BCa tumorigenesis and tumor metastasis were uncovered, the underlying molecular mechanism has not been thoroughly elucidated. Lacking of sophisticated understanding of the pathogenetic mechanism is one of the most crucial reasons for dismal outcomes in cancer patients [5]. Therefore, it is essential to explore new detailed mechanisms and molecular pathways activated in BCa for developing novel treatment options for anticancer therapy in BCa.

Transmembrane protein 40 (TMEM40) is a multi-pass membrane protein consisting of 233 amino acids and exists two isoforms [6–9]. It is localized at chromosome 3p25.2 and is believed to play a role in collagen induced arthritis (CIA) [10, 11]. A study reported that expression of TMEM40 were significantly higher in patients carrying Granulin (GRN) mutations compared with asymptomatic carriers and other frontotemporal lobar degeneration (FTLD) [12]. Importantly, our previous finding suggested that the expression of TMEM40 in BCa was significantly related to its pathologic grade, clinical stage, histological grade and pT status [13]. However, the specific function of TMEM40 in bladder carcinogenesis, especially its molecular mechanisms by which TMEM40 exhibits its functions and modulates the malignant behaviors of BCa cells, has not been fully understood.

In this study, we initially explored TMEM40 mRNA and protein expression and the correlation with malignant behavior, confirmed its potential role in proliferation, migration, and invasion of bladder cancer cells in vitro and tumorigenicity in vivo. Furthermore, we investigated the biologic functions of TMEM40 and underlying molecular mechanisms of BCa occurrence and progression in BCa cells.

## Methods

### Patients and clinical samples collection

BCa tissues and adjacent normal tissues were obtained from 12 patients, who underwent bladder surgical resection without preoperative systemic therapy in Nanfang Hospital of Southern Medical University between October 2014 and September 2016. After surgical removal, the tissues were immediately frozen using liquid nitrogen. All the patients signed informed consent and the study was approved by the Ethics Committee of Nanfang Hospital of Southern Medical University.

### Cell lines and cell culture

BCa EJ, J82, BIU-87, UMUC3, SW780, 5637, T24 and normal urothelial cell line SV-HUC-1 cells used in this study were obtained from laboratory preservation. The 5637, EJ, SW780, J82 and BIU87 cells were cultured in RPMI-1640 Medium (Invitrogen, Carlsbad, CA, USA), the UMUC3, T24 cells were cultured in Dulbecco’s Modified Eagle Medium (Invitrogen, Carlsbad, CA, USA), SV-HUC-1 cells were cultured in F12K (Invitrogen, Carlsbad, CA, USA). All cells were plus 10% fetal bovine serum (FBS) and 1% ampicillin (100 units/mL) and streptomycin (100 units/mL) and then placed at 37 °C with a humidified atmosphere of 95% air and 5% CO_2_ in incubator.

### siRNA or shRNA and plasmid DNA transfection

The specific small interfering RNA of TMEM40 and a scrambled negative control were purchased from RiboBio (Guangzhou, China). The overexpression vector pEZ-M98 and a scrambled negative control empty vector were also purchased from GeneCopoeia (Guangzhou, China). The shRNA vector and a scrambled negative control vector were obtained from Genechem (Shanghai, China). The sequence of siRNA was 5′-GGAUGAGCUUCAACUCUAUTT-3′; NC was 5′-UUCUCCGAACGUGUCACGUTT-3′; shRNA was 5′-GUGGACGCCUCUCAGUUAA-3′; NC was 5′-TTCTCCGAACGTGTCACGT-3′;

After cells were cultured 24 h, the cells were transiently transfected with corresponding siRNA or plasmid DNA using Lipofectamine 2000 reagents (Invitrogen, Carlsbad, CA, USA) according to the manufacturer’s instructions. After 48 h, cells transfected with siRNA or shRNA and plasmid were harvested for qRT-PCR or western blot detection.

### Real-time reverse transcription PCR (qRT-PCR)

All total RNA was extracted from BCa tissues or cells using the Trizol reagent (Invitrogen, Carlsbad, CA, USA). Qualified total RNA was then reverse transcribed to complementary DNA using a PrimeScript RT reagent Kit (Takara, Dalian, China). The quantitative real-time polymerase chain reaction (qRT-PCR) was performed using SYBR Green PCR kit (Takara, Dalian, China) following the manufacturer’s instructions. GAPDH was measured as an internal control. The primer sequences were listed as follows: TMEM40 primers: (forward, 5′-GCGGTAGGGGTGTACGGT-3′; reverse, 5′-CCGGACACGCTGAACT TGT-3′); P53 primers: (forward, 5-AAGATCCGCGGGCGTAA-3; reverse, 5′-CATCCTTTAACTCTAAG GCCTCATTC-3′); c-myc primers: (forward, 5′-CTTCTCTCCGTCCTCGGATTCT-3′; reverse, 5′-GAA GGTGATCCAGACTCTGACCTT-3′); JNK2 primers: (forward, 5′-TACGTGGTGACACGGTACTACC-3′; reverse, 5′-CACAACCTTTCACCAGCTCTCC-3′); RB primers: (forward, 5′-ATCAAGGGTCATTATGG GTTAGGC-3′; reverse, 5′-TAGGTGTAGGGGAGGGGAGAAGC-3′); GAPDH primers: (forward, 5′-CG CTCTCTGCTCCTCCTGTTC-3′; reverse, 5′-ATCCGTTGACTCCGACCTTCAC-3′). The reactions were carried out on an ABI PRISM 7500 Fluorescent Quantitative PCR System (Applied Biosystems, Foster City, CA, USA) in triplicate. The average value in each triplicate was used to calculate the relative amount of TMEM40 using 2^−ΔΔCt^ methods.

### Western blot analysis and antibodies

The BCa cells and tumor tissue samples lysates were extracted with RIPA cell lysis buffer, and the protein concentration in the lysates was quantified using an enhanced bicinchoninic acid protein assay kit (Thermo Fisher Scientific, MA, USA) with bovine serum albumin as a standard. The total protein extract will be used for western blot analysis. Equal amounts of total protein of tissues or cells were subjected to 10% SDS-PAGE and transferred to a PVDF membrane followed by immunoblotting using the following primary polyclonal antibodies: mouse anti-TMEM40 (Santa Cruz, CA, USA), rabbit anti-p53 (Proteintech Group, INC, USA), rabbit anti-p21 (Proteintech Group, INC, USA), mouse anti-CCND1 (Proteintech Group, INC, USA), rabbit anti-Caspase-3 (Cell Signaling Technology, MA, USA), rabbit anti-Caspase-9 (Proteintech Group, INC, USA), rabbit anti-PARP (Proteintech Group, INC, USA), rabbit anti-c-MYC (Proteintech Group, INC, USA) and GAPDH (Cell Signaling Technology, MA, USA). Specific proteins were detected with enhanced chemiluminescence (ECL, Millipore, USA). Band density was measured (ImageJ software) and normalized to GAPDH.

### Cell migration and invasion assay

A scratch wound healing assay was adapted to evaluate the ability of cell migration. Cells at a density of 80–90% confluence in 12-well plates were scratched manually with a sterile 200 µL plastic pipette tip, cultured for 24 h and photographed under a light microscope. The width of the wound area was quantitated, using the Image J. For the invasion assay, a transwell chamber was placed into a 24-well plate and was coated with 30 μL Matrigel and incubated for 60 min at 37 °C. Cells were cultured in normal medium and transfected with corresponding siRNA or plasmid DNA. 24 h after transfection, 5 × 10^4^ cells were first starved in 200 μL serum free medium and then placed in the uncoated dishes. The lower chamber was filled with 750 μL of complete medium. The cells were incubated for 48 h at 37 °C in a 5% CO_2_ incubator, and then the cells that had migrated to the bottom surface of the filter membrane were stained with 0.1% crystal violet staining solution and photographed in five preset fields per insert. The results represented the average of three independent experiments.

### Cell Counting Kit-8 assay

Cell proliferation was determined using Cell Counting Kit-8 (Beyotime Inst Biotech, China). In short, 5 × 10^3^ cells/well were seeded in a 96-well flat-bottomed plate for 24 h, then transfected with corresponding siRNA or plasmid DNA cultured in normal medium. At 0, 24, 48 and 72 h after transfection, 10 μL of CCK-8 (5 mg/mL) was added to each well and the cells were cultured for 2 h then determined the absorbance at a wavelength of 450 nm using a microplate reader (Tecan, Infinite^®^M200, Austria). Experiments were repeated at least three times.

### 5-Ethynyl-2′-deoxyuridine (EdU) incorporation assay

Cell proliferation was also determined by 5-Ethynyl-2′-deoxyuridine incorporation assay using an EdU Apollo DNA in vitro kit (RiboBio, Guangzhou, China) following the manufacturer’s instructions. Briefly, 5637 and EJ cells were plated in confocol dish plates at a density of 4 × 10^3^–1×10^5^ cells and incubated with 100 μL of 50 μM EdU per dish for 2 h at 37 °C, at 48 h after transfected with corresponding siRNA or plasmid DNA, respectively. Then, the cells were fixed for 30 min at room temperature using 100 μL of fixing buffer (4% polyformaldehyde containing PBS). Subsequently, the cells were incubated with 100 μL of 2 mg/mL glycine for 5 min followed by washing with 100 μL of PBS (Phosphate Buffered Saline). After permeabilization with 100 μL of 0.5% Triton X-100 for 10 min, the cells were reacted with 100 μL of 1× Apollo solution for 30 min at room temperature in the dark. After that, cells were incubated with 100 μL of 1× Hoechst 33342 solution for 30 min at room temperature in the dark followed by washing with 100 μL of PBS. The cells were then visualized under a fluorescence microscopy. Experiments were repeated at least three times.

### Cell cycle assay

The BCa cells were plated in 6-well plates at a density of 1 × 10^6^ cells every well cultured for 24 h, then transfected with siRNA and plasmid DNA. After 48 h, cells were then treated with trypsin, fixed in 70% frozen ethanol, followed by overnight incubation and washed with PBS, nucleus was stained by 450 μL Propidium Iodide (PI) and 50 μL RNAase. The percentages of 5637/EJ cells in the G0/G1, S, and G2/M phases were examined by Becton–Dickinson FACSVerse™ flow cytometry (San Jose, CA, USA). All experiments were performed three times.

### Cell apoptosis assay

Briefly, BCa cells were plated in 6-well plates at a density of 1–5×10^5^ cells every well cultured for 24 h. After transfection for 48 h, cells were harvested by trypsinization and washed with PBS and suspended in 500 μL Annexin V binding buffer. The percentage of cells actively undergoing apoptosis was determined by double staining with 5 μL AnnexinV-APC and 5 μL 7-AAD apoptosis detection kits (KeyGEN Biotech, Nanjing, China), and analyzed using a Becton–Dickinson FACSVerse™ flow cytometry (San Jose, CA, USA). At least 10,000 cells were obtained from each sample.

### Tumorigenicity assay in nude mice

Five-week-old female/male BALB/c-nu athymic nude mice (Experimental Animal Center of Southern Medical University, Guangzhou, China) were subcutaneously injected in the right flank with 1.0 × 10^7^ cells in 0.2 mL of PBS. Once tumors were formed, tumor volume (V) was measured daily by caliper and calculated using the formula V = (L × W^2^)/2, where L was the length and W was the width of the tumor. The mice were randomly divided into two groups (n = 6) for inoculation of shRNA transfected EJ cells and negative control (NC) for 27 days. Growth curves were plotted using average tumor volume within each experimental group every 3 days. 4 weeks later, the mice were euthanized, and the dissected tumors were collected. All animal experiments were approved by the animal center of the Southern Medical University.

### Statistical analysis

All statistical analyses were performed using the IBM SPSS 20.0 software package (SPSS, Inc., Chicago, IL, USA). Group difference was assessed using Student’s t test. Data are presented as the mean ± SD based on at least three repeats. Differences were considered to be statistically significant at P < 0.05.

## Results

### Expression of TMEM40 was upregulated in BCa tissues and cell lines compared with their normal entities

**Fig. 1:**
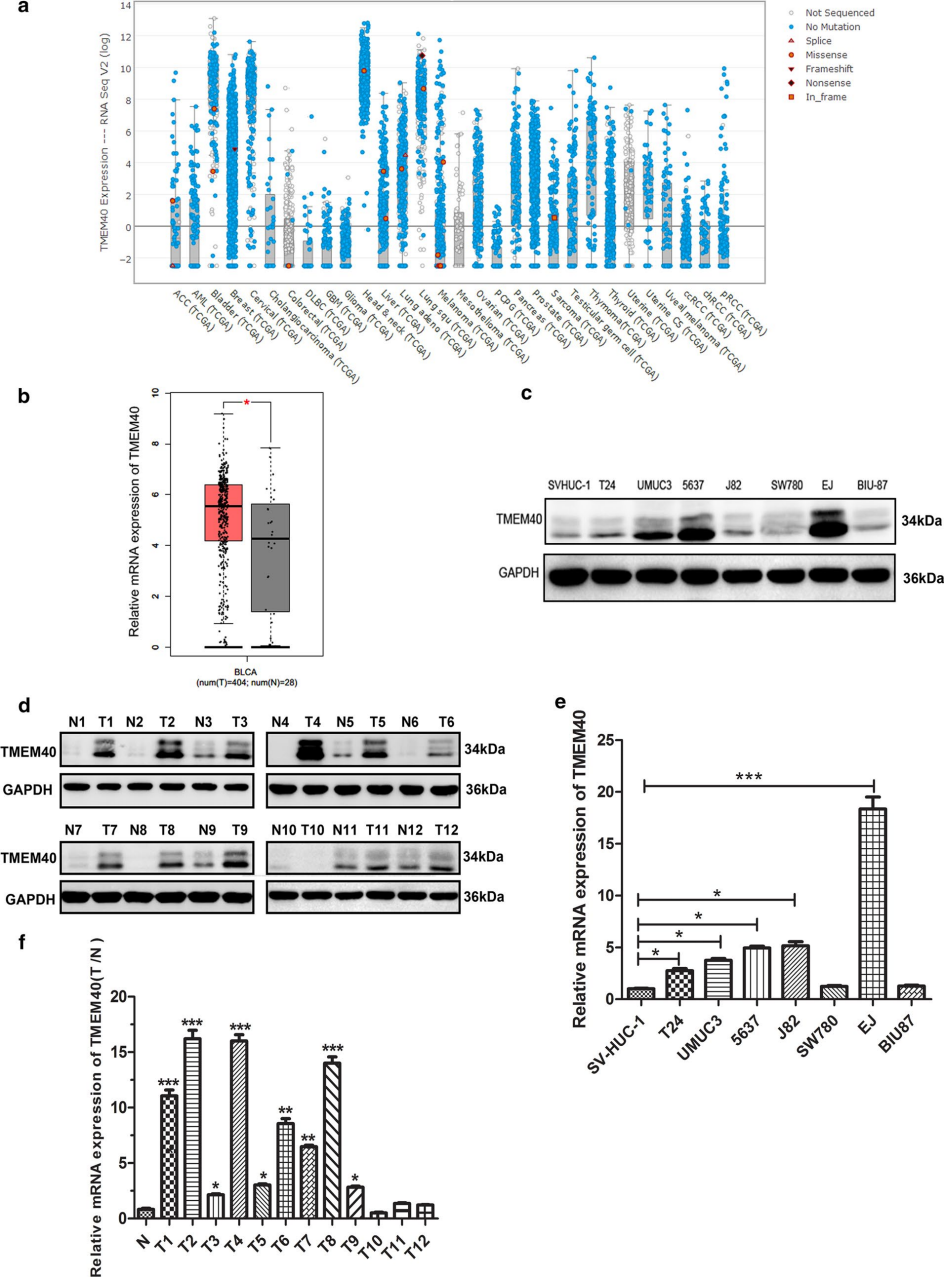
TMEM40 was overexpressed in bladder cancer tissues and cancer cells. **a** TMEM40 mRNA expression was significantly increased in bladder cancer and other organ cancers compared with their controls (n = 30). **b** Quantification of TMEM40 mRNA expression in bladder cancer and normal tissues. TMEM40 expression was significantly increased in bladder cancer tissues (n = 404) compared with normal tissues from the patients (n = 28) from the GEPIA dataset. Differences in TMEM40 expression levels between bladder cancer tissues and pair-matched noncancerous tissues. The expression of TMEM40 was normalized to that of GADPH. Statistical differences between samples were analyzed with paired samples t-test (n = 12). **d** Protein. **f** mRNA. Expression level of TMEM40 in seven BC cell lines T24, 5637, EJ, J82, UMUC3, SW780, BIU87 and normal urothelial cell line SVHUC-1 cells. **c** Protein. **e** mRNA. Data are presented as mean ± SD based on at least three independent experiments (*P < 0.05, **P < 0.01, ***P < 0.001)

From the expression of TMEM40 RNAseq data of the Cancer Genome Atlas (TCGA), we found that TMEM40 expression levels was significantly increased in BCa and other organ cancers compared with their controls (Fig. 1a, N = 30). Furthermore, from the Gene Expression Profiling Interactive Analysis (GEPIA), we also found that the level of TMEM40 in the tumor tissues (N = 404) was significantly higher than that in the controlled bladder tissues (N = 28) (Fig. 1b, *P* < 0.05). To investigate the expression status of TMEM40 in BCa, western blotting and quantitative RT-PCR analyses were performed in seven BCa cell lines (i.e. EJ, T24, 5637, SW780, UMUC3, J82 and BIU-87), one normal uroepithelial cell line SV-HUC-1 and 12 cases fresh BCa tissues paired with their adjacent non-neoplastic bladder tissues. The results showed that TMEM40 were significantly upregulated in five BCa cell lines (i.e. EJ, T24, 5637, UMUC3 and J82) compared with normal uroepithelial cell line SV-HUC-1 both on protein and mRNA levels (Fig. 1c, e, *P* < 0.05). Similarly, TMEM40 were considerably higher in BCa tissue specimens when compared with their paired normal bladder tissues (Fig. 1d, f, *P* < 0.05).

### TMEM40 knockdown in BCa cells transfected with siRNA against TMEM40 or it upregulation by pEZ-M98 vector

**Fig. 2:**
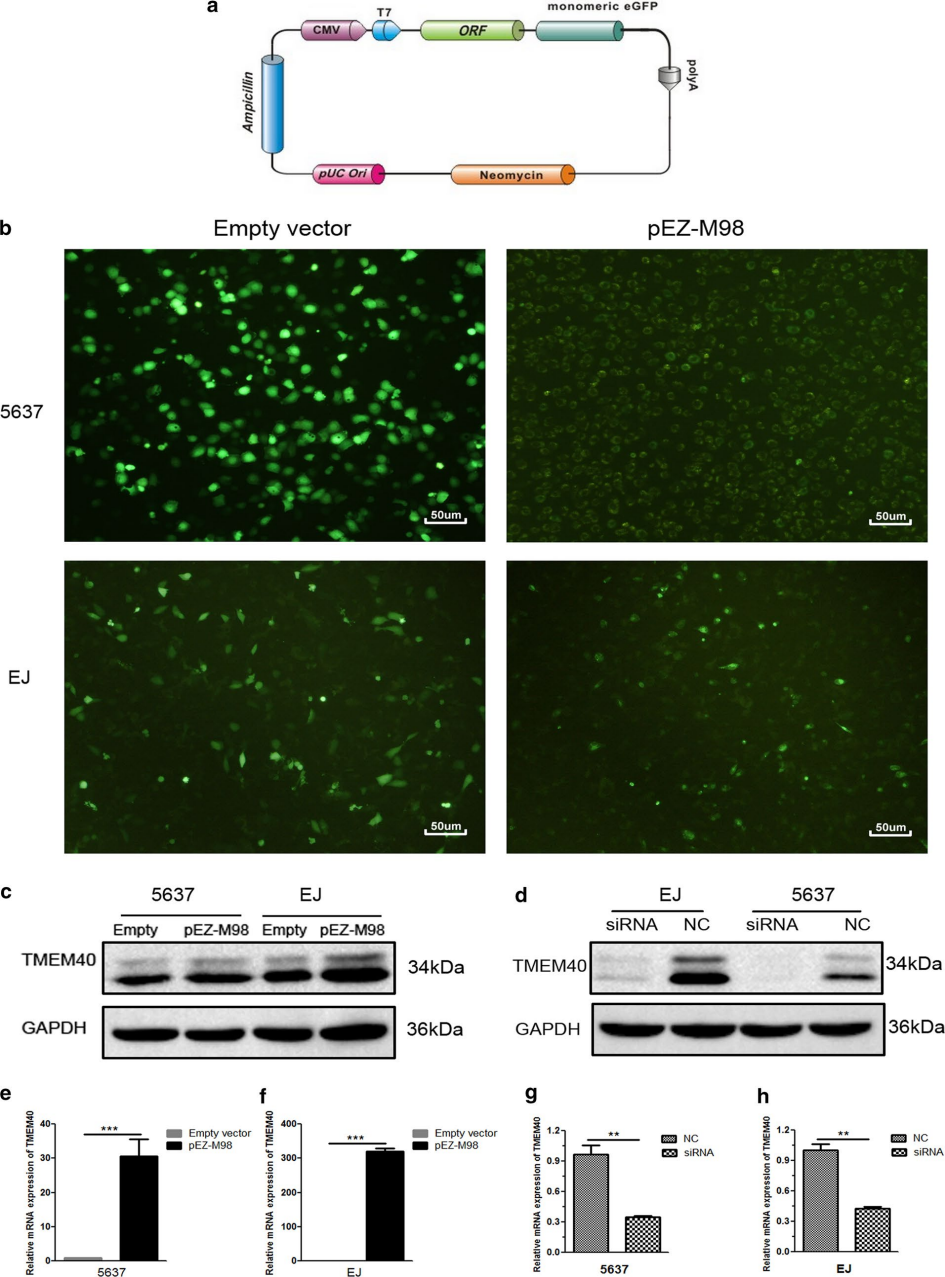
pEZ-M98-TMEM40-GFP vector construction and the expression of TMEM40. **a** The overexpression vector pEZ-M98-TMEM40 was engineered to express TMEM40 under the T7 promoter. **b** The expression of green fluorescent protein after 48 h of pEZ-M98 vector/empty vector transfection in 5637 and EJ cells (magnification ×100). The pEZ-M98 significantly upregulated the expression level of TMEM40 in 5637 and EJ cells. **c** Protein. **e**, **f** mRNA. The TMEM40-siRNA significantly down-regulated the expression level of TMEM40 in 5637 and EJ cells. **d** Protein. **g**, **h** mRNA (*P < 0.05, **P < 0.01, ***P < 0.001)

To investigate the functions of TMEM40 in BCa, overexpression vector containing green fluorescence protein (GFP) (Fig. 2a) were used to increase the expression of TMEM40 in 5637 and EJ cells. Then transfected with the vector and tested the efficiency of fluorescence expression (Fig. 2b). The data showed that pEZ-M98 vector could upregulate both TMEM40 mRNA (Fig. 2e, f) and protein (Fig. 2c) expressions in BCa cells. Meanwhile, we inhibited TMEM40 expression in BCa cells 5637 and EJ by transfecting them with its specific siRNA. Then we performed qRT-PCR and western blot to measure its expression levels at 48 h post-transfection. The data showed that TMEM40 expression could be effectively suppressed in BCa cells (Fig. 2d, g, h, *P* < 0.05).

### Silencing TMEM40 suppressed cell proliferation, migration and invasion, promoted cell apoptosis in BCa cells

**Fig. 3:**
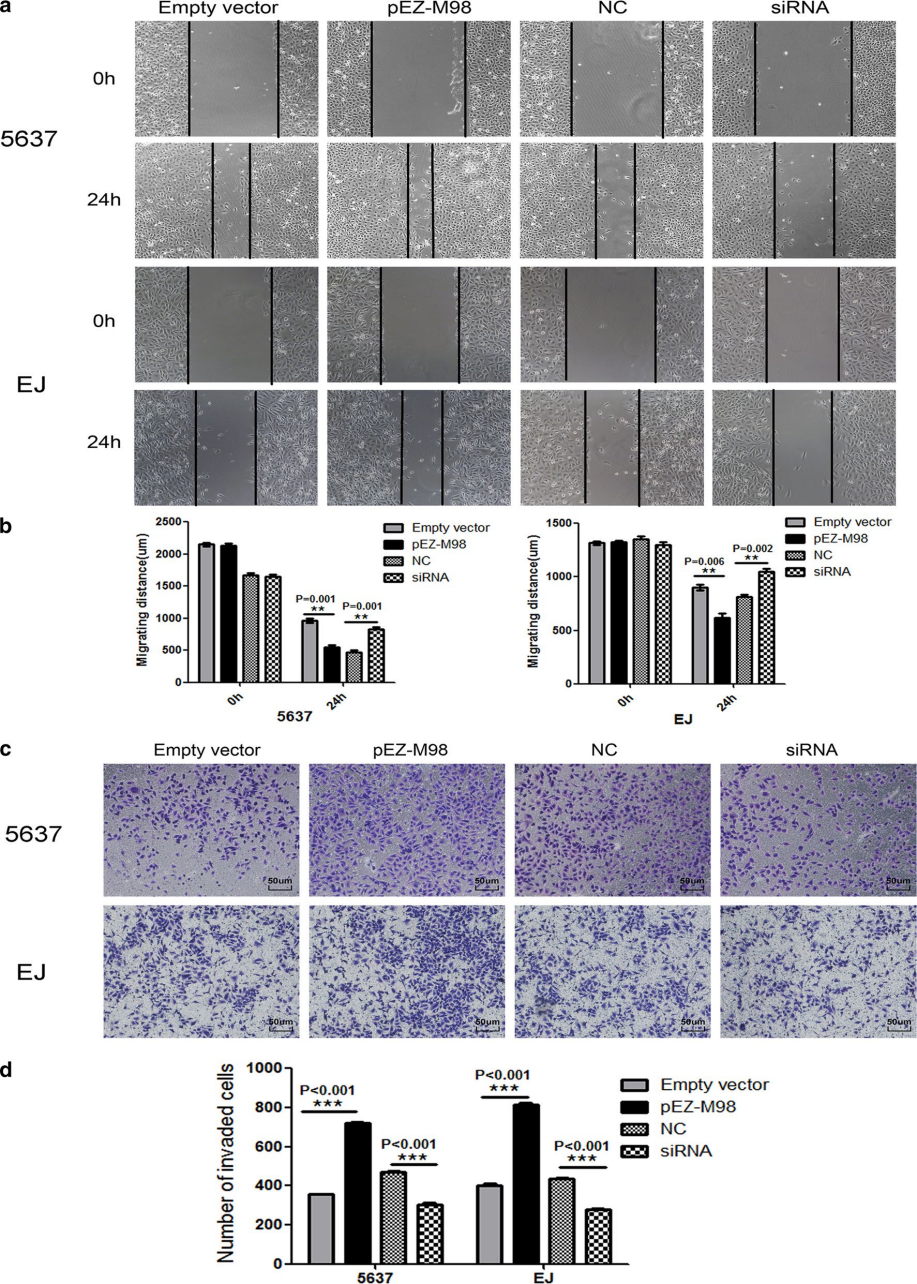
Overexpression of TMEM40 promotes bladder cancer cell migration and invasion. Cell migration and invasion was determined by wound healing and transwell assay. **a**, **b** Wound healing assay showed that TMEM40 shRNA resulted in a slower closing of scratch wounds was observed in bladder cancer 5637 and EJ cells. **c**, **d** Transwell invasion assay was measured and the results were expressed as the number of invaded cells per field compared with respective control. Data are expressed as the mean ± SD (magnification ×100, *P < 0.05, **P < 0.01, ***P < 0.001)

**Fig. 4:**
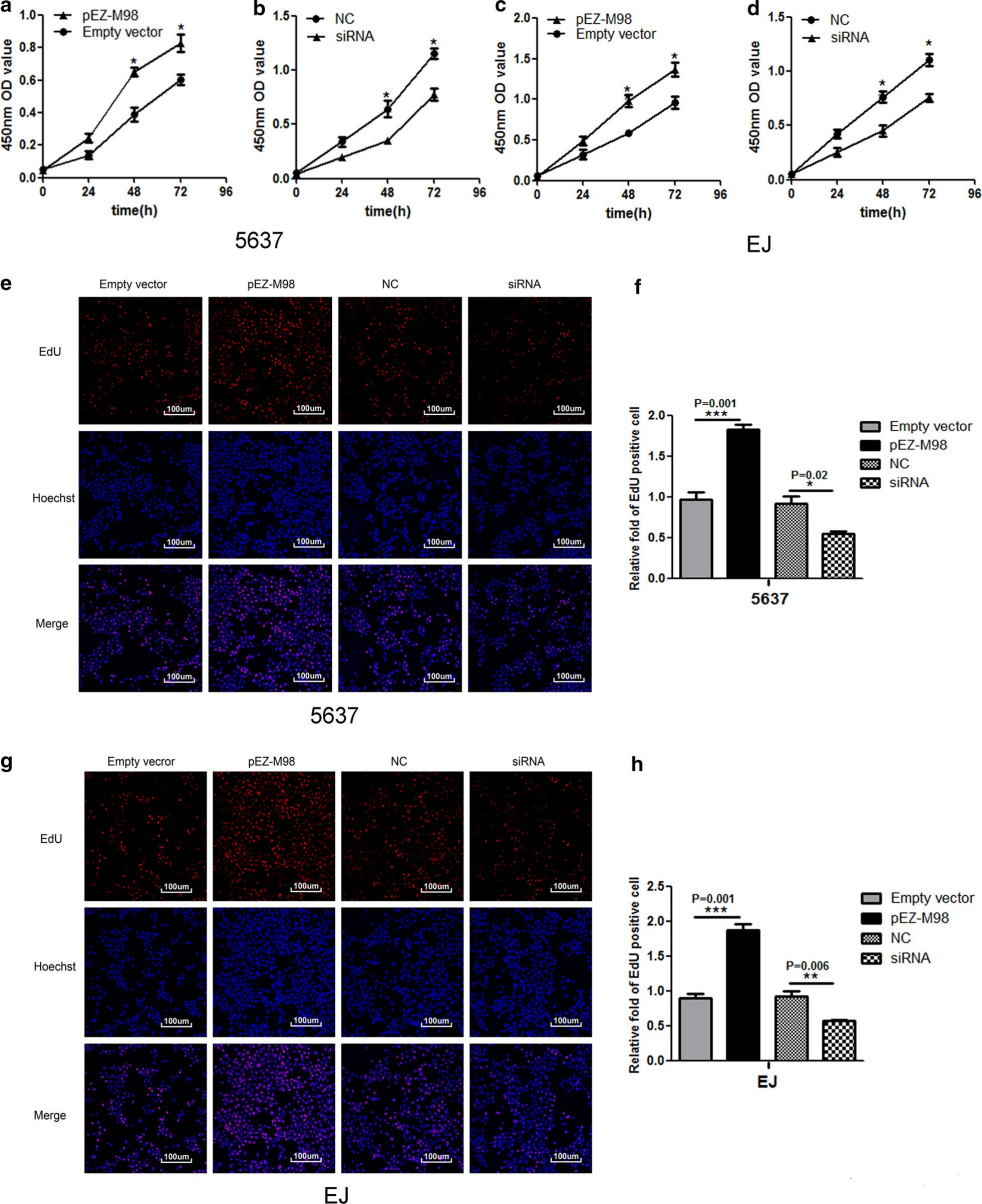
Effects of upregulation or downregulation of TMEM40 on cell proliferation in bladder cancer cells. Cell proliferation was determined by CCK-8 and EdU assay. Cell proliferation promotion was observed in bladder cancer 5637 cells (**a**), EJ cells (**c**). Cell proliferation inhibition was observed in bladder cancer 5637 cells (**b**), EJ cells (**d**). Whereas TMEM40 overexpression promote 5637 (**e**, **f**) and EJ cells (**g**, **h**) proliferation. Data are shown as mean ± SD (magnification ×100,*P < 0.05, **P < 0.01, ***P < 0.001)

**Fig. 5:**
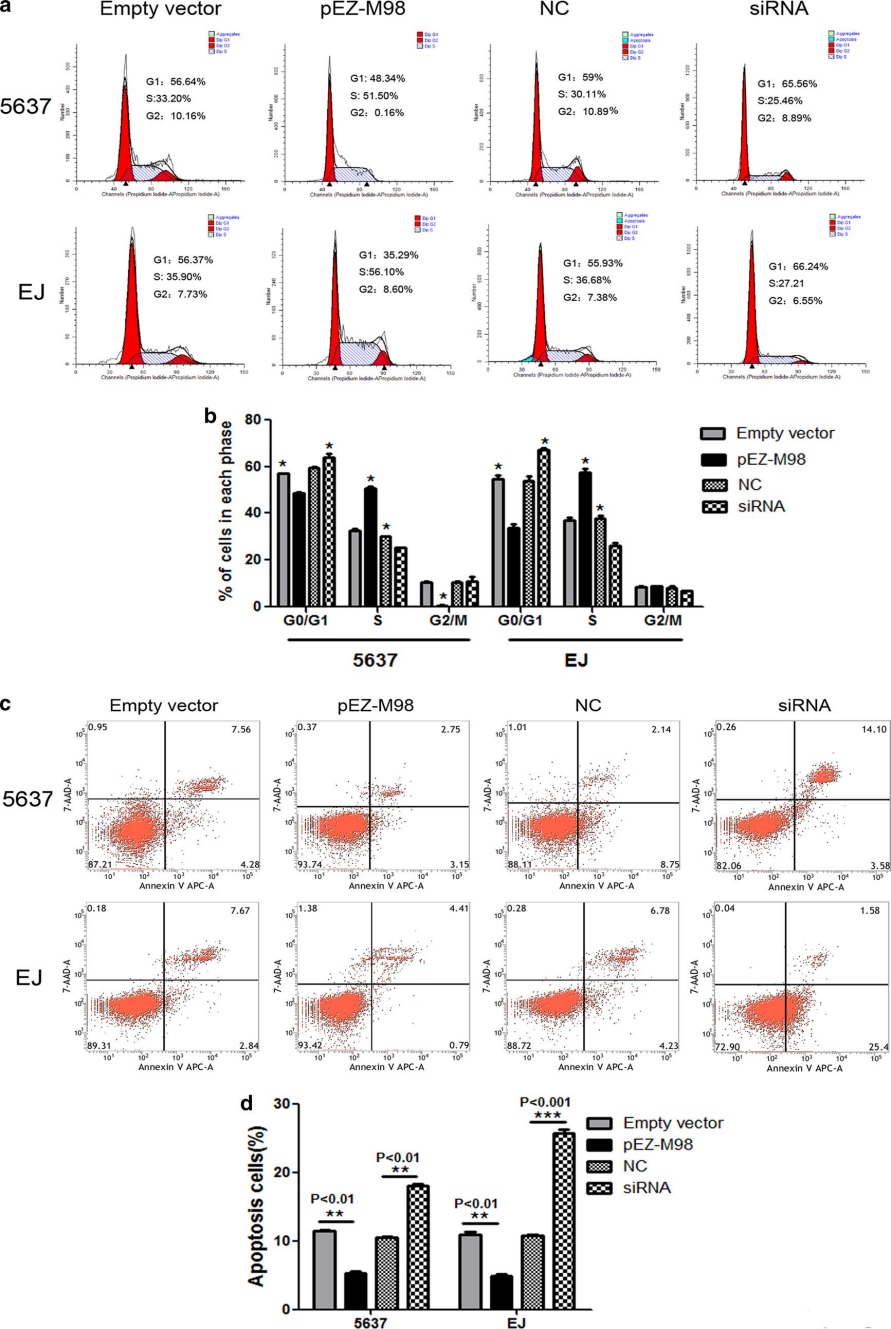
Overexpression of TMEM40 induced apoptosis and S phase cell cycle arrest in 5637 and EJ cells. Cell apoptosis and cell cycle was determined by flow cytometry. **a**, **b** Cell cycle analysis determined the relative cell numbers in each cell-cycle phase after propidium iodide staining of pEZ-M98 vector or siRNA 5637 and EJ cells. **c**, **d** Apoptotic cells increased significantly after 48 h transfection with siRNA compared to controls. Data represent the mean ± SD from three independent experiments (*P < 0.05, **P < 0.01, ***P < 0.001)

Based on the aforementioned results, we further evaluated whether TMEM40 could affect the biologic activities of BCa cells. Wound healing assay was used to assess the capability of cell motility, and the results showed that 5637 and EJ cells transfected with siRNA underwent a slower closing of scratch wounds compared with the negative control groups (Fig. 3a, b, *P* = 0.001; *P* = 0.002). Then transwell assay was applied to evaluate its impact on invasion ability in BCa cells, and the findings indicated that silencing TMEM40 significantly repressed the capability of invasion of BCa cells (Fig. 3c, d, *P* < 0.001). Meanwhile, the Cell Counting Kit-8 (CCK-8) (Fig. 4a–d, *P* < 0.05) and 5-Ethynyl-2′-deoxyuridine (EdU) (Fig. 4e–h, P < 0.05) assays demonstrated that suppression of TMEM40 attenuated cell proliferation in 5637 and EJ cells. Furthermore, results from the cell cycle analysis suggested that TMEM40 knockdown induced G1 phase arrest both in 5637 and EJ cells (Fig. 5a, b, *P* < 0.05). Finally, the effect of TMEM40 on cellular apoptosis was explored. FACS analysis showed that there was a significantly higher percentage of Annexin V-positive cells in TMEM40-silenced 5637 and EJ cells compared with control cells (Fig. 5c, d, *P* < 0.01). This indicated that TMEM40 knockdown induced cellular apoptosis. In contrast, overexpressing TMEM40 had proliferation, migration and invasion promoting effect as determined by the wound healing, CCK-8, EdU and transwell assay (Figs. 3, 4, 5, *P* < 0.05). From these results, we speculated that TMEM40 may play some important roles in BCa cells.

### Effects of TMEM40 on tumor growth in vivo and the expression of oncogenes and tumor suppressor genes

**Fig. 6:**
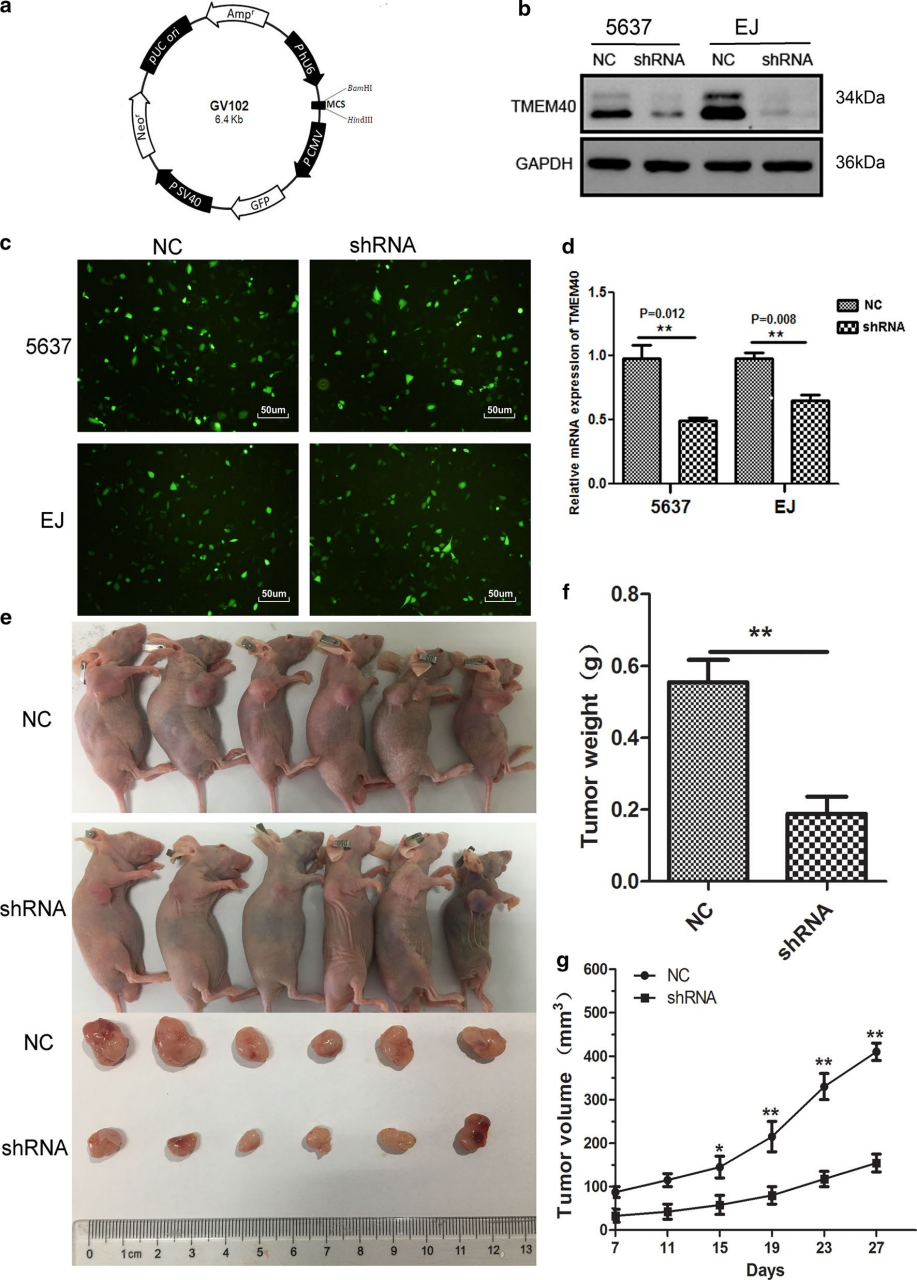
TMEM40 shRNA suppresses the expression of TMEM40 in bladder cancer cells in vitro and tumorigenicity in vivo. **a** The knockdown vector hU6-MCS-CMV-GFP-SV40-Neomycin was engineered to express shRNA under the U6 promoter. **c** The expression of green fluorescent protein after 48 h of shRNA vector/NC vector transfection in 5637 and EJ cells (magnification ×100). The shRNA significantly downregulated the expression level of TMEM40 in 5637 and EJ cells. **b** Protein. **d** mRNA. **e** Representative images of tumorigenicity assay performed in nude mice. **f** Tumor weight was measured after sacrifice at the end of the experiment. **g** Tumor volume was monitored by caliper measurements twice weekly. All results were presented as the mean ± SD from 3 independent experiments (*P < 0.05, **P < 0.01, ***P < 0.001)

**Fig. 7:**
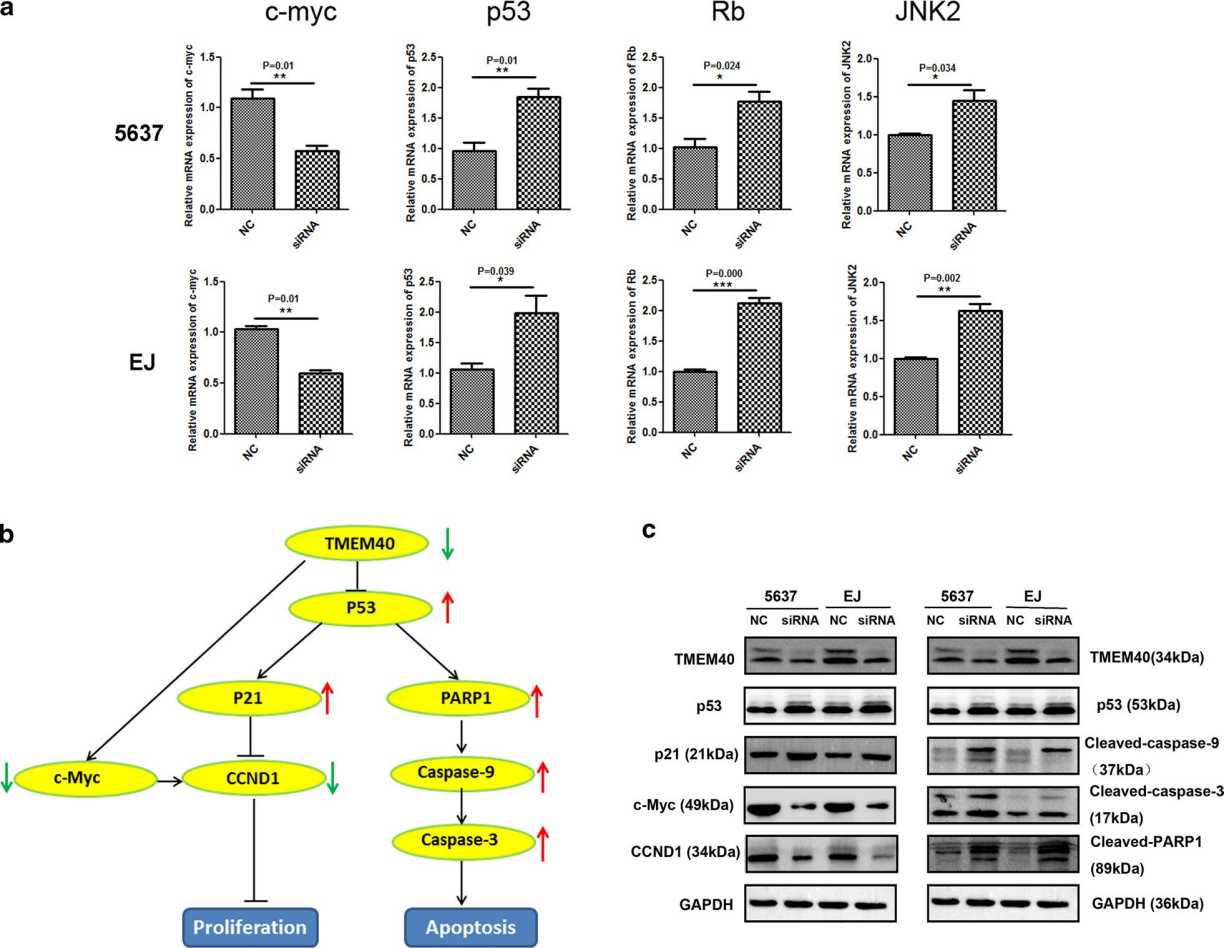
Effects of TMEM40 inactivation on the expression of oncogenes and tumor suppressor genes and knockdown of TMEM40 accelerates p53 and influenced the regulatory proteins of proliferation and apoptosis. **a** Quantitative RT-PCR of c-MYC, p53, Rb and JNK2 in 5637/EJ-NC and 5637/EJ shRNA cells. Expression of each specific gene was normalized to that of GAPDH. Transcription amount is presented relative to that of EJ/5637 control line. **b** The working model of TMEM40-regulated bladder tumor progression and development. **c** Western blotting analysis of proliferation-related signals and apoptosis-related signals in bladder cancer cells. Data are shown as mean ± SD (*P < 0.05, **P < 0.01, ***P < 0.001)

The short hairpin RNA (shRNA) (Fig. 6a) was introduced into 5637 and EJ cell lines, which exhibit low TMEM40 expression, whereas exogenous TMEM40 was stably introduced into 5637 cell, which shows relative high TMEM40 expression (Fig. 6b–d, *P* < 0.05). To confirm the oncogenic efficiency of TMEM40 in vivo, we constructed a BALB/c nude mouse xenograft model by EJ cells. Our results demonstrated the tumor weight and volume of tumors in nude mice treated with shRNA were markedly suppressed (38.5% of decrease in tumor weight) relative to that of treated with empty vector (Fig. 6f, *P* < 0.05). These data indicated that knockdown of TMEM40 markedly inhibited the tumorigenicity of EJ cells in the nude mouse xenograft model (Fig. 6e, g, *P* < 0.05). Moreover, we checked the expression levels of oncogenes (i.e. c-myc), as well as tumor suppressor genes (i.e. p53, Rb and JNK2), all of which were well known to be involved in urothelial tumorigenesis, in NC versus siRNA cells. TMEM40 silencing resulted in significant downregulation of the expression of c-myc as well as significant upregulation of the expression of p53, Rb and JNK2 (Fig. 7a, *P* < 0.05).

### TMEM40 acted as an oncogene via regulating the p53 pathway

To comprehensively find mechanistic insight into the role of TMEM40 in regulating BCa cell proliferation and apoptosis, we measured the alteration of proliferation-related signals (p53, p21, c-MYC, cyclin D1) and apoptosis-related proteins (p53, activated caspase-3, activated caspase-9, activated PARP) by western blotting. Knockdown of TMEM40 increased the expression levels of p53 and p21, activated caspase-3, caspase-9, and PRAP and decreased the levels of c-MYC and cyclin D1 in 5637 and EJ cells. Together, all the above suggested that TMEM40 knockdown reduced the proliferation and enhanced apoptosis of BCa cells (Fig. 7b, c, *P* < 0.05).

## Discussion

BCa is one of the most common urinary malignancies worldwide [14]. Most patients are diagnosed at advanced stage of BCa because patients with BCa are lack of specific symptoms at early stage [15–18]. Nevertheless, the poor understanding of BCa development mechanism has limited the effective adjuvant therapies for BCa [19–21].

To the best of our knowledge, the biological functions of TMEM40 in BCa have not been characterized yet. In the present study, we found that TMEM40 expression was significantly up-regulated in BCa tissues and cells compared with non-neoplastic urothelium. We identified that TMEM40 overexpression is a characteristic molecular change in BCa and explore the biological roles of TMEM40 on the phenotypes of BCa cells in vitro. To investigate the TMEM40 biological functions in BCa, we used CCK8, EdU, scratch assays, transwell and apoptosis assays to detect cell proliferation, migration, invasion and apoptosis, respectively. TMEM40 silencing results in inhibition of BCa cell proliferation, migration, invasion and induced cell apoptosis. Furthermore, TMEM40 knockdown also blocked G1-to-S cell cycle transition and these effects could be reversed by overexpressed it in BCa cells.

In mouse xenograft models for BCa, TMEM40 knockdown was also found to considerably retard tumor formation and subsequent tumor growth [22]. Moreover, TMEM40 knockdown was associated with downregulated expression of oncogenes (i.e. c-myc) and upregulated expression of tumor suppressor genes (i.e. p53, Rb and JNK2) in BCa cells [23]. These findings in conjunction with our in vitro data suggested that TMEM40 played important roles in the malignant phenotype regulation of BCa and likely to promote bladder tumorigenesis and tumor progression.

The tumor suppressor p53 is a transcription factor that regulates several cellular stress responses, including DNA repair, metabolism, cell cycle arrest, genomic stability, apoptosis and senescence, through induction of the various transcriptions of target genes [24–26]. We finally investigated the levels of proliferation-related signals (i.e. p53, p21, c-MYC, cyclin D1) and apoptosis-related proteins (i.e. p53, activated caspase-3, activated caspase-9, activated PARP) by western blotting. Our results revealed that knockdown of TMEM40 increased the levels of p53, p21, activated caspase-3, caspase-9, and PRAP and decreased the levels of c-MYC, cyclin D1 in 5637 and EJ cells [27–29]. These findings suggest that TMEM40 was a proto-oncogene which could promote BCa development and progression via the p53 signaling pathway in BCa.

## Conclusions

Taken together, our findings provide evidence that TMEM40 play oncogenic roles in the development and progression of BCa through the p53 signaling pathway. And our data gives a more comprehensive understanding of how TMEM40 functions in BCa and demonstrates its potential as a powerful therapeutic approach to treat BCa.

## Abbreviations

TMEM40: transmembrane protein 40
BCa: bladder cancer
FBS: fetal bovine serum
GRN: granulin
DMEM/F12: Dulbecco’s Modified Eagle Medium/Nutrient Mixture F-12
qRT-PCR: quantitative real-time PCR
PVDF: polyvinylidene fluoride
CCK-8: Cell Counting Kit-8
EdU: 5-ethynyl-2′-deoxyuridine
NC: negative control
siRNA: small interfering RNA
SDS-PAGE: sodium dodecyl sulfate polyacrylamide gel electrophoresis
shRNA: short hairpin RNA
RPMI-1640: Roswell Park Memorial Institute 1640
FTLD: frontotemporal lobar degeneration
CIA: collagen induced arthritis
PARP: poly(ADP-ribose) polymerase
GAPDH: glyceraldehyde-3-phosphate dehydrogenase
GFP: green fluorescence protein
PBS: phosphate buffered saline
